# Ammonia Stress Disrupts Intestinal Health in *Litopenaeus vannamei* Under Seawater and Low-Salinity Environments by Impairing Mucosal Integrity, Antioxidant Capability, Immunity, Energy Metabolism, and Microbial Community

**DOI:** 10.3390/antiox14111383

**Published:** 2025-11-20

**Authors:** Yafei Duan, Yuxiu Nan, Jitao Li, Meng Xiao, Yun Wang, Ruijie Zhu

**Affiliations:** 1 State Key Laboratory of Mariculture Biobreeding and Sustainable Goods, Key Laboratory of South China Sea Fishery Resources Exploitation & Utilization, Ministry of Agriculture and Rural Affairs, South China Sea Fisheries Research Institute, Chinese Academy of Fishery Sciences, Guangzhou 510300, China; 2 Yellow Sea Fisheries Research Institute, Chinese Academy of Fishery Sciences, Qingdao 266071, China

**Keywords:** shrimp, intestine, ammonia, salinity, immunity, microbiota

## Abstract

Ammonia is a key water quality factor limiting shrimp aquaculture. Intestinal health is closely associated with the nutrition, metabolism and immunity of shrimp. However, the response characteristics of the shrimp intestine to ammonia stress under seawater and low-salinity environments remain unclear. In this study, the shrimp *Litopenaeus vannamei* reared in seawater (salinity 30) or low-salinity (salinity 3) water were subjected to ammonia stress for 14 days, respectively. The changes in intestinal morphology, antioxidant capacity, immune response, energy metabolism, and microbial community were systematically investigated. The results showed that ammonia stress induced intestinal tissue damage in both seawater and low-salinity cultured shrimp, characterized by epithelial cell detachment and mucosal structural disruption. At the molecular level, ammonia stress triggered intestinal stress responses by interfering with key physiological processes such as antioxidant defense and endoplasmic reticulum stress. This process further led to varying degrees of disorders in physiological functions, including immune regulation, inflammatory response, and autophagic activity. In addition, ammonia stress disrupted the homeostatic balance of intestinal energy metabolism by affecting the expression of genes related to glucose metabolism, the tricarboxylic acid (TCA) cycle, and mitochondrial respiratory chain. In addition, ammonia stress increased the diversity of intestinal microbiota and caused microbial dysbiosis by increasing harmful bacteria (e.g., *Vibrio*) and decreasing beneficial bacterial groups (e.g., *Bacillus*). Ammonia stress generally enhanced intestinal microbiota chemotaxis. Specifically, predicted functions of microbiota in seawater-cultured shrimp showed increased carbohydrate, linoleic acid, and cofactor/vitamin metabolism; in low-salinity-cultured shrimp, functions including protein digestion/absorption, flavonoid/steroid hormone biosynthesis, and glycosaminoglycan degradation were reduced. These results revealed that ammonia stress compromised shrimp intestinal health by disrupting mucosal structure, triggering stress responses, and disturbing immune function, energy metabolism, and microbial homeostasis. Notably, low-salinity cultured shrimp exhibited more pronounced intestinal stress responses and greater physiological vulnerability than seawater-cultured counterparts.

## 1. Introduction

The Pacific white shrimp, *Litopenaeus vannamei*, is a key species for the global aquaculture industry, making significant contributions to the fisheries economy. This shrimp species can tolerate a wide range of salinities, surviving in aquatic environments with a salinity of 0.5–40 [1]. With the increasing intensification of shrimp farming, higher feed input has frequently led to elevated ammonia concentrations in culture water, inducing recurrent environmental stress [2]. This has become a key factor causing stress-related mortality in shrimp. Previous studies have shown that ammonia stress can cause histopathological damage, induce stress responses, weaken immune capacity, and disrupt metabolic functions, ultimately threatening the survival and growth of *L. vannamei* [3,4,5,6,7,8]. In addition to regulating water quality, maintaining intestinal health homeostasis of shrimp usually achieves an ideal effect in preventing environmental stress.

Oxidative stress is a major contributing factor to shrimp mortality induced by ammonia stress. After ammonia enters a shrimp’s body, it interferes with cellular metabolism and mitochondrial function, leading to the production of large amounts of free radicals such as reactive oxygen species (ROS) in the organism. This disrupts the dynamic balance of the oxidative system, thereby inducing oxidative stress. This state induces oxidative damage to essential biomacromolecules in shrimp, including lipids, proteins, and DNA, which in turn leads to cellular dysfunction, tissue damage, and ultimately affects their growth, immunity, and even survival [2]. In addition, ammonia stress disrupts the normal energy metabolic balance of shrimp. On one hand, to counteract physiological disorders (e.g., oxidative stress and immunosuppression) induced by ammonia toxicity, shrimp need to allocate substantial energy to activate defense and repair mechanisms, leading to a sharp increase in basal energy consumption. On the other hand, ammonia stress may directly interfere with core intracellular metabolic pathways of shrimp, resulting in reduced energy production efficiency. This contradiction between “increased demand” and “impaired supply” forces the organism to reallocate energy resources, often at the expense of growth and immunity.

The intestine is a key organ for nutrient metabolism and immune defense, and is crucial to shrimp health [9]. However, the shrimp intestine is highly vulnerable to environmental stress, which can lead to pathological damage and functional impairment. Several studies have reported on the impacts of ammonia stress on intestinal homeostasis in shrimp. For example, ammonia stress could compromise the integrity of the intestinal mucosa in *L. vannamei*, causing stress responses, immune dysregulation, and physiological disturbances [3]. Ammonia stress could trigger ROS overproduction in the intestine of *L. vannamei*, progressing to endoplasmic reticulum (ER) stress and mitochondrial fission, with the resultant damage ultimately mediated by enhanced apoptosis and suppressed cell proliferation, leading to intestinal inflammation and injury [10].

The intestine harbors a vast microbiota that contributes to the host’s health homeostasis by participating in core physiological processes such as nutrient metabolism, immune regulation, and barrier defense. The intestinal microbiota plays a critical role in maintaining intestinal microecological balance, regulating host immunity and metabolism, which is closely linked to shrimp health status [11,12,13]. The rapid development of 16S rRNA gene sequencing has facilitated the analysis of microbial diversity and composition, enabling its application in studies investigating the impact of ammonia stress on intestinal microbiota homeostasis in shrimp. It was reported that ammonia stress could reduce intestinal microbial diversity and disrupt the balance between beneficial and pathogenic bacteria [3,4,14,15], thereby facilitating the enrichment of intestinal pathogens [16]. Ammonia stress could also enhance the functions related to biofilm formation, host colonization, and virulence of the intestinal microbiota of *L. vannamei* [17]. Additionally, ammonia stress could disrupt intestinal metabolism of *L. vannamei*, including amino acids, pyrimidines, purines, and alkaloids [3]. However, the mechanisms of ammonia stress that affect the intestinal health of shrimp cultured under low-salinity and seawater conditions remain poorly understood.

Therefore, in this study, following a 14-day ammonia exposure under seawater or low-salinity conditions, the toxic impact of ammonia stress on intestinal health homeostasis was investigated in *L. vannamei* through a multi-dimensional assessment of the intestinal barrier. The specific aspects examined included: (1) the histomorphological changes in the intestinal mucosa; (2) the alterations in intestinal physiological functions, including antioxidant defense, ER stress, antimicrobial activity, inflammation, and autophagy; (3) the changes in intestinal metabolic functions, including glycolysis, TCA cycle, and respiratory chain; (4) the shifts in the diversity, composition, and functional profile of the intestinal microbiota. The results can provide a theoretical foundation for understanding how ammonia stress induces intestinal toxicity in shrimp under both seawater and low-salinity culture conditions.

## 2. Materials and Methods

### 2.1. Experimental Animals and Rearing Conditions

Healthy *L. vannamei* shrimp, averaging 9.9 ± 0.2 g in body weight, were reared under two conditions: seawater (salinity 30) and low-salinity water (salinity 3). Among them, the low-salinity cultured shrimp were derived from their seawater counterparts through a 27-day gradual salinity desalination process. The detailed procedure was as follows: All shrimp originated from the same parent stock, and they were co-reared in an indoor pond at a salinity of 30, starting from the post-larval 10 (PL10) stage. Twenty-seven days prior to the stress experiment, the shrimp reared in seawater (salinity 30) were randomly divided into two ponds. One pond was maintained salinity of 30 (seawater group), while the other underwent a gradual reduction in salinity to 3 (low-salinity group). This was achieved by using aerated tap water to lower the salinity by 1 unit per day over the 27-day period. Prior to the ammonia exposure, a 7-day acclimation period was implemented in the experimental tanks to allow the shrimp to stabilize. Throughout the temporary rearing stage, key water quality parameters were maintained as follows: temperature at 26.0 ± 0.5 °C, pH between 7.9 and 8.1, and dissolved oxygen (sustained by continuous aeration) above 5 mg/L. Daily management included a 50% water exchange. Shrimp were administered a commercial diet at 5% of their body weight, with residues and feces being promptly removed. The commercial compound feed for shrimp was purchased from Guangdong Yuequn Ocean Biotechnology Co., Ltd. (Jieyang, China), and its main nutritional composition is crude protein ≥ 44.0%, crude fat ≥ 6.0%, crude fiber ≤ 5.0%, crude ash ≤ 18.0%, moisture ≤ 10.0%, lysine ≥ 2.5%, and total phosphorus 0.9% ≥ 1.5%.

### 2.2. Ammonia Stress Exposure and Sample Collection

**Figure 1:**
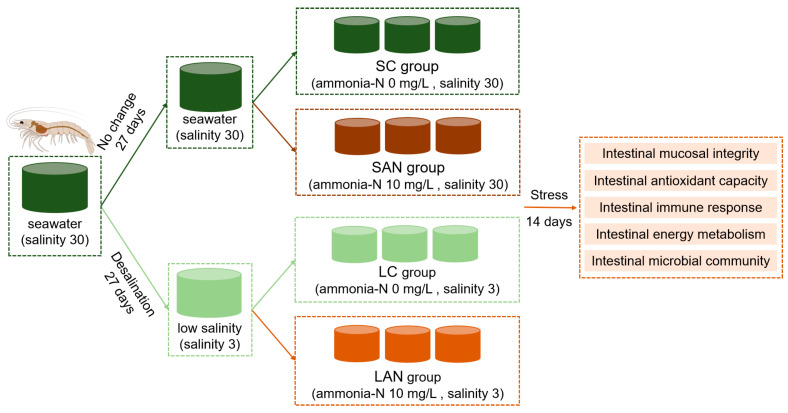
Schematic diagram of the experimental design. The same color indicates the same experimental environment, i.e., the culture water is identical; different colors represent different stress exposure conditions.

Following the 7-day acclimation, the shrimp were allocated into four experimental groups: the seawater control (SC), seawater ammonia stress (SAN), low-salinity control (LC), and low-salinity ammonia stress (LAN) groups. Each treatment was assigned to three replicate tanks, with 30 shrimp per tank. Based on preliminary experiments and existing research reports, the ammonia-N concentration for stress treatment in this study was set at 10 mg/L. Except for differences in the salinity and ammonia-N concentration of the rearing water, all the other rearing conditions were consistent across the four groups. Specifically, the SC group was maintained in water at salinity 30 with 0 mg/L ammonia-N; the SAN group at salinity 30 with 10 mg/L ammonia-N; the LC group at salinity 3 with 0 mg/L ammonia-N; and the LAN group at salinity 3 with 10 mg/L ammonia-N. After calculation, the average proportion of un-ionized ammonia (NH_3_) in this study was 5.74–5.75%, with a concentration of 0.57–0.58 mg/L. The ammonia-N concentration was adjusted using ammonium chloride and was monitored and regularly adjusted to maintain a stable level. The ammonia-N concentration of the rearing water was measured using the hypobromite oxidation method. Half of the rearing water in each tank was replaced daily with pre-prepared water that had been adjusted to the corresponding salinity and ammonia nitrogen concentration. Water quality during the stress period was maintained identically to the acclimation period, with the exception of the target ammonia-N and salinity levels. During the ammonia stress exposure period, the ammonia-N concentration was basically stable at the set level, nitrite-N remained below 0.05 mg/L within the safe range, the temperature was 26.0 ± 0.5 °C, the pH was 7.9–8.1, and dissolved oxygen was above 5 mg/L. The stress exposure experiment lasted for 14 days. The study design is presented in Figure 1.

The sampling was conducted following the 14-day ammonia stress exposure. Intestines from five shrimp per tank were pooled in RNAFollow reagent (NCM Biotech, Suzhou, China) for gene expression analysis; another set of intestines from five shrimp was pooled for intestinal microbiota analysis. Additionally, the intestines from three shrimp were fixed in 4% paraformaldehyde for histological morphology analysis.

### 2.3. Intestinal Histomorphological Analysis

Following fixation, intestinal tissues were processed through a series of steps: they were washed, dehydrated through a graded ethanol series, and cleared with xylene. The samples were then embedded in paraffin and sectioned into 4 μm slices. Finally, the sections were stained with H&E, air-dried at room temperature, and mounted with neutral resin for preservation. Histological morphology was subsequently examined and imaged under a light microscope.

### 2.4. Gene Expression Analysis

Total RNA was extracted from shrimp intestinal tissues using Trizol reagent. The RNA integrity was verified by 1.0% agarose gel electrophoresis. Furthermore, RNA purity and concentration were determined using a spectrophotometer (NanoDrop2000, Thermo Scientific, Shanghai, China), with all the samples exhibiting A_260_/A_280_ ratios between 1.8 and 2.0. Gene-specific primers were designed, and the *β*-actin gene was used as the internal control (Table S1). The SYBR Green Pro Taq HS Premix kit (Accurate Biotechnology Co., Ltd., Changsha, China) was adopted for real-time quantitative PCR (qPCR) operation in a real-time fluorescence quantitative PCR testing apparatus (Likang CG-02, Shanghai, China). The quantitative real-time PCR reaction mixture amount was 15 μL, comprising 7.5 μL SYBR Green Pro Taq HS Premix (2×), 5.3 μL RNase-free water, 0.6 μL 10 μmol/L forward primer, 0.6 μL 10 μmol/L reverse primer, and 1.0 μL cDNA. The qPCR reaction program was 95 °C for 30 s, 40 cycles of 95 °C for 5 s, and 60 °C for 30 s. The relative gene expression levels in each group were normalized to those of the SC group, and calculated using the method of Livak and Schmittgen [18]. The detailed experimental procedure was described in Nan et al. [19].

### 2.5. Intestinal Microbiota Analysis

Genomic DNA was extracted from intestinal microbiota samples and used as a template to amplify the V4 region of the 16S rDNA gene with PCR using universal primers. Following library preparation, sequencing was conducted on an Illumina platform. The resulting paired-end reads were merged and demultiplexed based on their unique barcodes. Subsequently, the sequences were clustered into operational taxonomic units (OTUs) at a 97% similarity threshold, with chimeric sequences removed. The α-diversity, including ACE, Chao1, Simpson, and Shannon indices, was calculated. The β-diversity was evaluated using the Principal Component Analysis (PCA) plot. The relative abundance of bacterial taxa was examined at the phylotypic levels of phylum and genus. LEfSe analysis was used to identify bacterial biomarker taxa. The bacterial correlation network was constructed based on the OTUs’ abundance using Cytoscape software v3.9.1. Predicted metabolic functions of the intestinal microbiota were analyzed using the KEGG database with RandomForest package v4.6-14 in R version 3.4.4 software. The detailed experimental procedure was described in Duan et al. [3].

### 2.6. Statistical Analysis

All the gene expression data were expressed as the mean ± standard error (SE). Statistical analysis was based on the mean values of each culture tank, where *n* represents the number of culture tanks, and each sample analysis was performed with more than three triplicate samples. Data normality and homogeneity of variances were assessed prior to the analysis using the Shapiro–Wilk and Levene’s tests, respectively. Statistical significance was determined using a two-way analysis of variance (ANOVA) followed by a post hoc test when significant interactions were detected. A *p*-value < 0.05 was considered statistically significant.

## 3. Results

### 3.1. Changes in Intestinal Tissue Morphology

**Figure 2:**
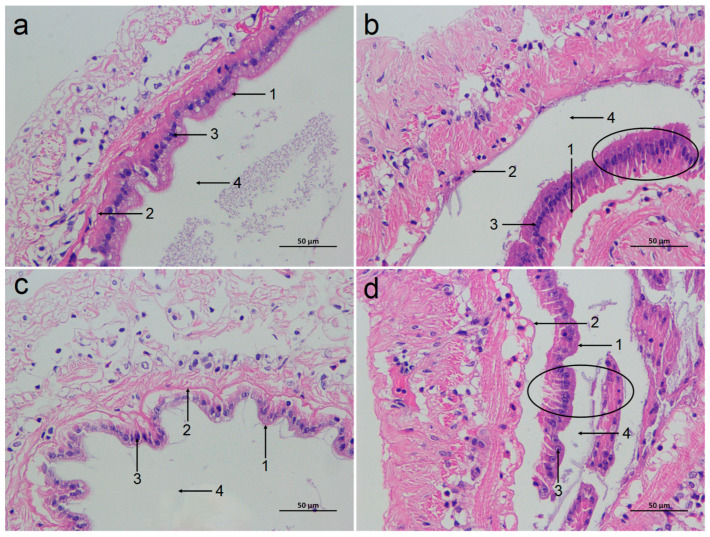
Effect of ammonia exposure on the intestinal histological morphology of *L. vannamei* reared in seawater and low-salinity environments. (**a**) The SC group. (**b**) The SAN group. (**c**) The LC group. (**d**) The LAN group. 1: brush border; 2: basement membrane; 3: nuclei; 4: lumen; 400× magnification. Circles indicate the shed intestinal mucosa.

Based on the HE staining, the tissue morphology of the intestinal mucosa of the SC group and LC groups was relatively normal, with no obvious damage (Figure 2a,c). However, the intestinal mucosa of both ammonia-stressed groups (SAN and LAN) showed severe damage, characterized by the detachment and disintegration of epithelial cells from the basement membrane, with cellular debris scattered into the lumen (Figure 2b,d).

### 3.2. Changes in Intestinal Stress Response Indicators

**Figure 3:**
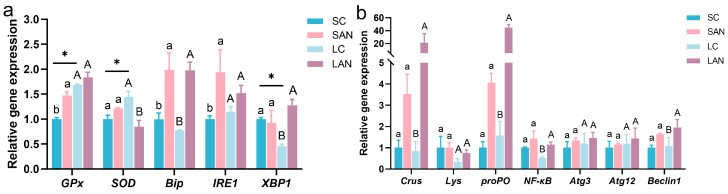
Effects of ammonia exposure on the stress response and immune gene expression in the intestine of *L. vannamei* reared in seawater and low-salinity environments. (**a**) Oxidative and endoplasmic reticulum stress-responsive genes. (**b**) Antimicrobial, inflammatory, and autophagy-related genes. Different letters (lowercase for salinity 30; uppercase for salinity 3) indicate significant differences (*p* < 0.05) between the two groups within each salinity; asterisk (*) indicates a significant difference between SC and LC groups (*p* < 0.05).

Compared with the SC group, antioxidant genes, such as the relative expression levels of glutathione peroxidase (*GPx*), were significantly increased in the SAN group; the expression of superoxide dismutase (*SOD*) was slightly increased without statistical significance. ER stress-related genes, such as the expression of immunoglobulin binding protein (*Bip*) and inositol demand enzyme 1α (*IRE1*), were significantly increased in the SAN group, while the expression of X-frame binding protein 1 (*XBP1*) gene was decreased with no statistical significance. Compared with the LC group, the LAN group exhibited up-regulation in the expressions of *GPx*, *Bip*, *IRE1* and *XBP1* genes, and down-regulation in the expression of *SOD* gene, but only the changes in *SOD*, *Bip* and *XBP1* were statistically significant. Furthermore, the LC group showed significantly elevated expressions of the *GPx* and *SOD* genes but a reduced expression of the *XBP1* gene relative to the SC group. (Figure 3a).

### 3.3. Changes in Intestinal Immunity, Inflammation, and Autophagy Indicators

Compared with the SC group, immune-related genes, such as the relative expression levels of crustin (*Crus*) and prophenoloxidase (*proPO*), inflammatory gene nuclear factor kappa B (*NF-κB*), and autophagy-related proteins 3 and 12 (*Atg3*, *Atg12*) and *Beclin1* genes were all increased in the SAN group, but none of these increases showed significant differences. Compared with the LC group, the expressions of *Crus*, lysozyme (*Lys*), *proPO*, *NF-κB*, *Atg3*, *Atg12* and *Beclin1* genes were all increased in the LAN group, and among these, only the changes in *Crus*, *proPO*, *NF-κB* and *Beclin1* genes were statistically significant (Figure 3b).

### 3.4. Changes in Intestinal Energy Metabolism-Related Indicators

#### 3.4.1. Carbohydrate Metabolism

**Figure 4:**
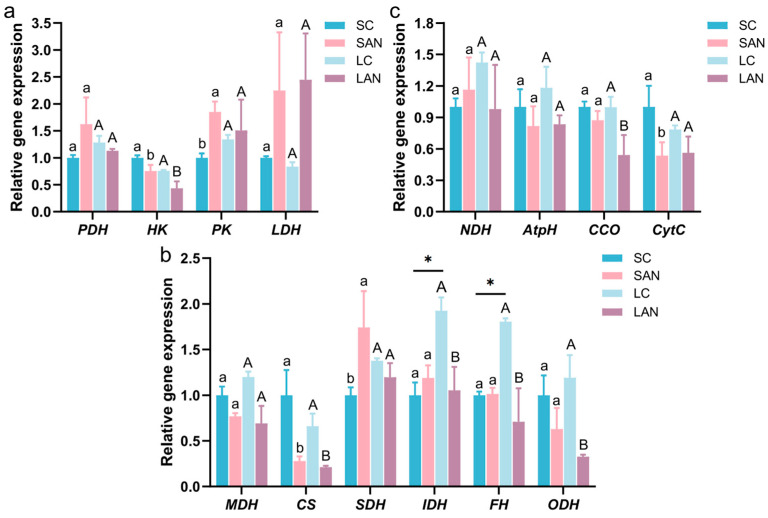
Effect of ammonia exposure on the energy metabolism-related gene expression in the intestine of *L. vannamei* reared in seawater and low-salinity environments. (**a**) Carbohydrate metabolism-related genes. (**b**) Tricarboxylic acid (TCA) cycle-related genes. (**c**) Respiratory chain-related genes. Different letters (lowercase for salinity 30; uppercase for salinity 3) indicate significant differences (*p* < 0.05) between the two groups within each salinity; asterisk (*) indicates a significant difference between SC and LC groups (*p* < 0.05).

Compared with the SC group, the SAN group exhibited the upregulation of pyruvate dehydrogenase (*PDH*), pyruvate kinase (*PK*), and lactate dehydrogenase (*LDH*) gene expression, while conversely, the hexokinase (*HK*) gene was downregulated. However, only the changes in the *HK* and *PK* genes showed significant differences. Compared with the LC group, the LAN group showed decreased expressions of *PDH* and *HK* genes but increased expressions of *PK* and *LDH* genes. Among these changes, only the change in the *HK* gene was statistically significant (Figure 4a).

#### 3.4.2. TCA Cycle

Compared with the SC group, the SAN group exhibited the downregulation of the relative expression levels of malate dehydrogenase (*MDH*), citrate synthase 1 (*CS*), and oxoglutarate dehydrogenase (*ODH*) genes, while conversely, the succinate dehydrogenase (*SDH*) and isocitrate dehydrogenase (*IDH*) genes were upregulated. However, only the changes in *CS* and *SDH* genes showed significant differences. Compared with the LC group, the expressions of *MDH*, *CS*, *SDH*, *IDH*, fumarase (*FH*), and *ODH* genes were all decreased in the LAN group, but among these changes, only the variations in *CS* and *SDH* genes were not statistically significant. In addition, the LC group exhibited a significant increase in the expression of *IDH* and *FH* genes compared to the SC group (Figure 4b).

#### 3.4.3. Respiratory Chain

Compared with the SC group, the SAN group exhibited the upregulation of NADH dehydrogenase (*NDH*) gene expression, while conversely, the ATP synthase (*AtpH*), cytochrome c oxidase (*CCO*), and cytochrome C (*CytC*) genes were downregulated. However, only the *CytC* gene change showed a significant difference. Compared with the LC group, the LAN group exhibited a reduction in the expressions of *NDH*, *AtpH*, *CCO*, and *CytC* genes, and among these changes, only the CCO gene change was statistically significant (Figure 4c).

### 3.5. Alterations in the Intestinal Microbiota

#### 3.5.1. Changes in Intestinal Microbial Diversity

**Figure 5:**
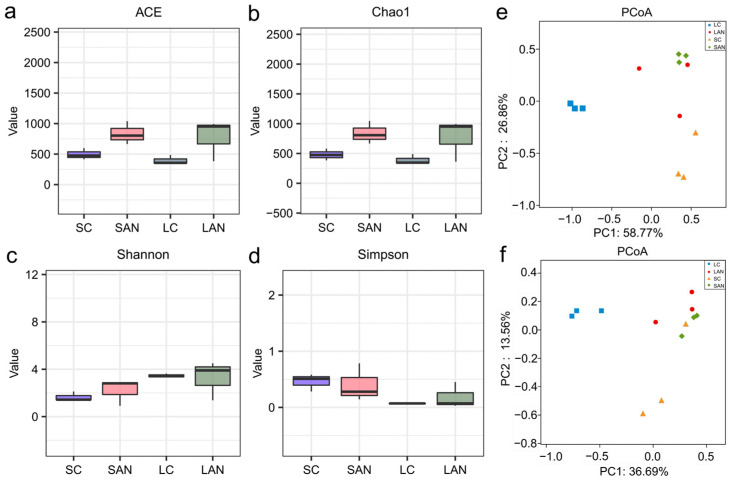
Effect of ammonia exposure on the intestinal microbiota diversity of *L. vannamei* reared in seawater and low-salinity environments. (**a**) ACE index. (**b**) Chao1 index. (**c**) Shannon index. (**d**) Simpson index. (**e**) PCoA plot based on the weighted UniFrac distance. (**f**) PCoA plot based on the unweighted UniFrac distance.

Compared with the SC group, the ACE, Chao1, and Shannon indices in the SAN group were increased, while the Simpson index was decreased. Compared with the LC group, the ACE, Chao1, and Shannon indices in the LAN group were all increased, whereas the Simpson index showed no obvious change (Figure 5a–d). The β-diversity changes in intestinal microbiota were analyzed based on the NMDS plot. It can be observed that the intestinal microbiota patterns of the four groups were significantly different and could be clearly separated (Figure 5e,f).

#### 3.5.2. Changes in Intestinal Microbiota Composition

**Figure 6:**
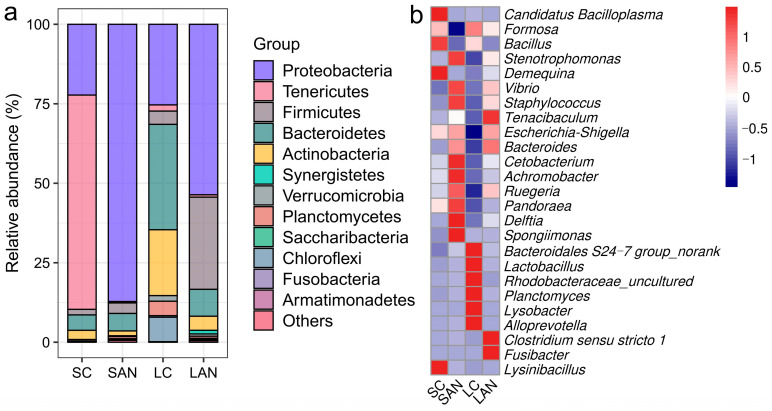
Effect of ammonia exposure on the intestinal microbiota composition of *L. vannamei* reared in seawater and low-salinity environments. (**a**) Relative abundance of bacterial phyla. (**b**) Heatmap of the relative abundance of bacterial genera. Red indicates high abundance, and blue represents low abundance.

The composition of the intestinal bacterial community was analyzed across different taxonomic levels. At the phylum level, the SAN group showed increased relative abundances of Proteobacteria, Firmicutes, Bacteroidetes, and Synergistetes but decreased abundances of Actinobacteria and Tenericutes when compared to the SC group. Relative to the LC group, the LAN group exhibited higher abundances of Proteobacteria, Firmicutes, and Synergistetes, in contrast to marked reductions in Bacteroidetes, Actinobacteria, Tenericutes, Verrucomicrobia, Planctomycetes, and Chloroflexi (Figure 6a).

At the genus level, compared with the SC group, the SAN group exhibited an increase in the relative abundances of *Vibrio*, *Escherichia-Shigella*, *Pandoraea*, *Achromobacter*, *Ruegeria*, *Delftia*, *Bacteroidales S24-7 group*, *Lactobacillus*, *Rhodobacteraceae_uncultured*, *Tenacibaculum*, *Bacteroides*, *Planctomyces*, *Cetobacterium*, *Staphylococcus*, and *Spongiimonas*, but a decrease in *Candidatus Bacilloplasma*, *Formosa*, *Demequina*, *Bacillus*, *Lysinibacillus*, and *Stenotrophomonas*. Compared with the LC group, the LAN group exhibited an increase in the relative abundances of *Vibrio*, *Escherichia-Shigella*, *Pandoraea*, *Demequina*, *Stenotrophomonas*, *Achromobacter*, *Ruegeria*, *Delftia*, *Tenacibaculum*, *Bacteroides*, *Cetobacterium*, *Clostridium sensu stricto 1*, *Staphylococcus*, *Fusibacter*, and *Spongiimonas*, but a decrease in *Candidatus Bacilloplasma*, *Formosa*, *Bacillus*, *Bacteroidales S24-7 group*, *Lactobacillus*, *Rhodobacteraceae_uncultured*, *Planctomyces*, *Alloprevotella*, and *Lysobacter* (Figure 6b).

#### 3.5.3. Identification of Differential Taxa in the Intestinal Microbiota

**Figure 7:**
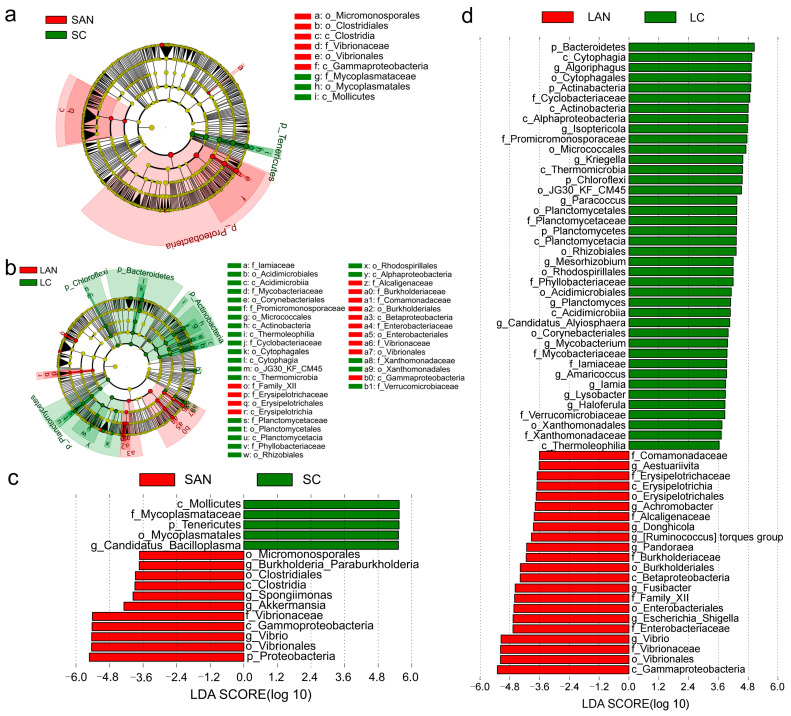
The intergroup variations in the intestinal microbiota of *L. vannamei* reared in seawater and low-salinity environments. The LEfSe cladogram of the seawater cultured shrimp (**a**) and low-salinity cultured shrimp (**b**). The LDA score of the seawater cultured shrimp (**c**) and low-salinity cultured shrimp (**d**).

Differential intestinal bacterial taxa associated with the stress response were identified by LEfSe analysis. As shown in the cladogram, the family Mycoplasmataceae was enriched in the SC group; the family Vibrionaceae was enriched in the SAN group (Figure 7a). The families Iamiaceae, Mycobacteriaceae, Promicromonosporaceae, Cyclobacteriaceae, Planctomycetaceae, Phyllobacteriaceae, Xanthomonadaceae, and Verrucomicrobiaceae were enriched in the LC group; the families Erysipelotrichaceae, Alcaligenaceae, Burkholderiaceae, Comamonadaceae, Enterobacteriaceae, and Vibrionaceae were enriched in the LAN group (Figure 7b).

Several bacterial genera exhibited significant enrichment with an LDA score greater than 3.0: The genus *Candidatus Bacilloplasma* was predominantly enriched in the SC group; the genera *Vibrio*, *Akkermansia*, *Spongiimonas*, and *Burkholderia Paraburkholderia* were predominantly enriched in the SAN group (Figure 7c). The genera *Haloferula*, *Lysobacter*, *Iamia*, *Amaricoccus*, *Mycobacterium*, *Candidatus Alysiosphaera*, *Planctomyces*, *Mesorhizobium*, *Paracoccus*, *Kriegella*, *Isoptericola*, and *Algoriphagus* were enriched in the LC group; *Vibrio*, *Escherichia-Shigella*, *Fusibacter*, *Pandoraea*, *Ruminococcus torques group*, *Donghicola*, *Achromobacter*, and *Aestuariivita* were significantly enriched in the LAN group (Figure 7d).

#### 3.5.4. The Correlation Network of Intestinal Bacteria

**Figure 8:**
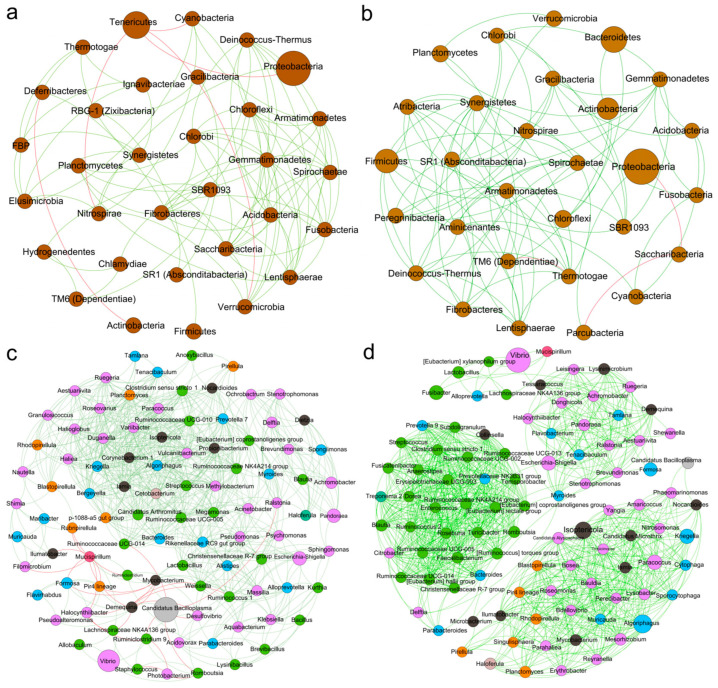
The correlation network of the intestinal microbiota of *L. vannamei* reared in seawater and low-salinity environments. The correlation network of the bacterial phyla of the seawater cultured shrimp (**a**) and low-salinity cultured shrimp (**b**). The correlation network of the bacterial genera of the seawater cultured shrimp (**c**) and low-salinity cultured shrimp (**d**). Circular nodes represent bacterial phyla or genera. Lines between them indicate correlations, with green for positive correlations and red for negative correlations.

We constructed a correlation network to explore the interactions among intestinal bacteria. At the phylum level, in the seawater cultured shrimp, Proteobacteria was negatively correlated with Tenericutes; Deferribacteres exhibited a positive correlation with Firmicutes but had a negative correlation with Actinobacteria. Furthermore, a significant negative correlation was identified between Tenericutes and Verrucomicrobia (Figure 8a). In the low-salinity cultured shrimp, Proteobacteria were negatively correlated with Saccharibacteria; Firmicutes were positively correlated with Synergistetes and Fibrobacteres; Actinobacteria were positively correlated with Bacteroidetes and Planctomycetes; and Bacteroidetes were positively correlated with Chloroflexi (Figure 8b).

At the genus level, the intestinal microbial network of the low-salinity cultured shrimp was more complex than that of the seawater cultured shrimp, presenting three clustered communities. In seawater-cultured shrimp, *Vibrio* exhibited a positive correlation with *Staphylococcus*; *Tenacibaculum* exhibited a positive correlation with *Cetobacterium*; *Lactobacillus* exhibited a positive correlation with *Bacteroides* and *Alloprevotella*; *Bacteroides* was positively correlated with *Cetobacterium*; *Alloprevotella* was positively correlated with *Escherichia-Shigella*; and *Demequina* was positively correlated with *Candidatus Bacilloplasma* (Figure 8c). In the low-salinity cultured shrimp, *Tenacibaculum*, *Achromobacter*, *Escherichia-Shigella*, and *Demequina* exhibited positive correlations with each other; *Lactobacillus* exhibited a positive correlation with *Alloprevotella*; and Demequina exhibited a positive correlation with *Tenacibaculum* (Figure 8d).

#### 3.5.5. Changes in the Metabolic Functions of Intestinal Microbiota

**Figure 9:**
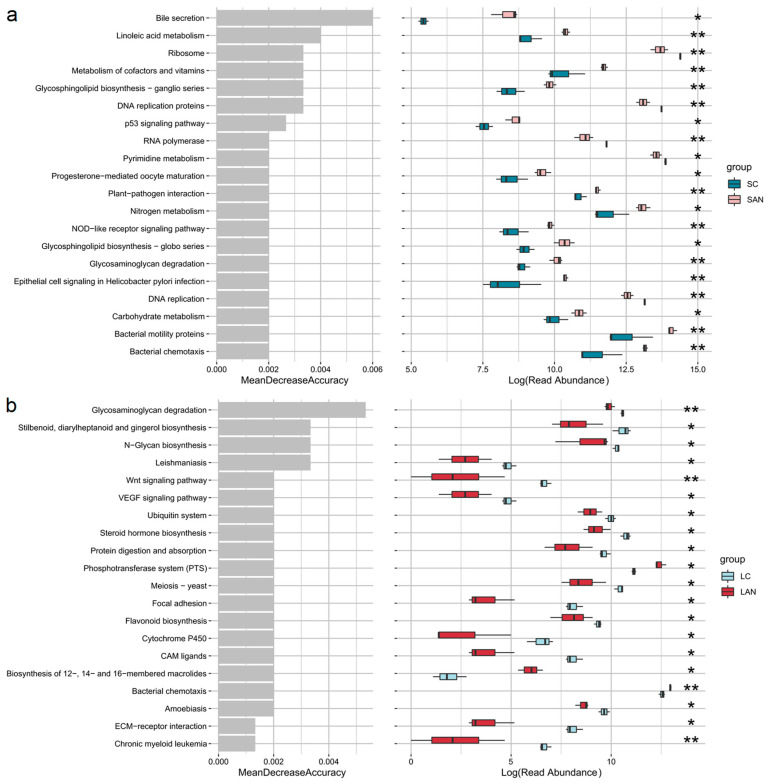
The top 20 predicted functions with significant differences in the intestinal microbiota of *L. vannamei* reared in seawater and low-salinity environments. (**a**) Changes in the metabolic functions of intestinal microbiota of the seawater-cultured shrimp. (**b**) Changes in the metabolic functions of intestinal microbiota of the low-salinity-cultured shrimp. Significance levels are marked with asterisks: * for *p* < 0.05 and ** for *p* < 0.01.

The changes in metabolic functions of the shrimp intestinal microbiota were analyzed. Compared with the SC group, the functions of “bacterial chemotaxis”, “bacterial motility proteins”, “carbohydrate metabolism”, “glycosaminoglycan degradation”, “glycosphingolipid biosynthesis—globo and ganglio series”, “nitrogen metabolism”, “metabolism of cofactors and vitamins”, “linoleic acid metabolism”, and “bile secretion” were significantly increased in the SAN group (Figure 9a). Compared with the LC group, the functions of “bacterial chemotaxis” and “phosphotransferase system (PTS)” were significantly increased in the LAN group, while “flavonoid biosynthesis”, “protein digestion and absorption”, “steroid hormone biosynthesis”, “ubiquitin system”, “N-glycan biosynthesis”, “stilbenoid, diarylheptanoid and gingerol biosynthesis”, and “glycosaminoglycan degradation” were significantly decreasd (Figure 9b).

## 4. Discussion

The intestine serves as a vital digestive and immune organ for shrimp, and its physiological status directly impacts the overall health and stress resistance of the organism [20]. It is reported that ammonia stress can damage the intestinal mucosa of *L. vannamei* [3]. Similarly, in this study, the intestinal mucosa of the seawater and low-salinity cultured shrimp both showed shedding and damage after ammonia stress, indicating that the physical barrier function of the intestine was impaired. These changes will inevitably affect the overall homeostasis of intestinal health of the shrimp.

Environmental stress induces oxidative stress in shrimp, mobilizing their antioxidant enzymes like GPx and SOD, to regulate redox balance [21]. In this study, *GPx* gene expression was elevated in the intestines of the shrimp cultured at both salinity levels under ammonia stress. In contrast, *SOD* expression was increased in the seawater group but decreased in the low-salinity group. This differential expression indicated that ammonia stress induced an oxidative stress response in the shrimp intestine, and the antioxidant defense system of the low-salinity cultured shrimp might be more severely disrupted, resulting in a diminished capacity to counteract oxidative damage compared to the seawater-cultured shrimp. Environmental stress induces ER stress, which regulates cellular adaptation through the unfolded protein response (UPR), where IRE1, Bip, and XBP1 serve as key proteins [22]. In this study, following ammonia stress, the ER stress markers *Bip* and *IRE1* were concurrently up-regulated in the shrimp intestine under both culture conditions. In contrast, the expression of *XBP1* was elevated in a salinity-dependent manner, showing an increase only in the low-salinity group. These findings collectively indicated that ammonia stress induced ER stress in the shrimp intestine under both culture conditions. The low-salinity cultured individuals mounted a more active and orchestrated adaptive protection mechanism, as evidenced by the specific activation of the complete IRE1-XBP1 signaling pathway to counteract ER dysfunction.

The intestinal immune system provides a crucial barrier for shrimp against stress. Crus and Lys are common antimicrobial molecules in shrimp, while proPO system is a vital component of shrimp immunity [5,23]. In this study, ammonia stress induced the up-regulation of *Crus* and *proPO* gene expression in the intestines of both seawater and low-salinity cultured shrimp, with the *Lys* gene showing a differential pattern of only slight elevation exclusive to the low-salinity group. This indicated that ammonia stress activated the innate immune response in the intestine, with *Crus* and *proPO* genes playing central roles under both environmental conditions, while *Lys* gene might contribute to specific immune regulation in the low-salinity condition. NF-κB plays a pivotal role as a transcriptional regulator, orchestrating the expression of a wide array of inflammatory factors [24]. Autophagy plays a critical role in cellular adaptation to environmental stress by clearing damaged intracellular components and maintaining substance and energy homeostasis. Atg3, Atg12 and Beclin1 are important molecules in the autophagy process [25]. In this study, the expressions of *NF-κB*, *Atg3*, *Atg12*, and *Beclin1* genes showed an upward trend in the intestines of the shrimp from both salinity groups following ammonia stress. These coordinated changes collectively suggested that ammonia stress might activate inflammatory and autophagy responses in the shrimp intestine. This suggested that the cells might coordinately initiate immune defense and self-clearing mechanisms to maintain homeostasis under stress conditions.

Cellular stress is a highly energy-consuming process. Glucose metabolism is a central pathway for energy production. Within this pathway, PDH serves as the key link between glycolysis and the TCA cycle; HK and PK act as rate-limiting enzymes of glycolysis; and LDH regulates the fate of glycolytic end-products by catalyzing the reversible interconversion of pyruvate and lactate [19,26]. In this study, ammonia stress increased *LDH* gene expression and decreased *HK* gene expression in the intestine of the shrimp from both the seawater and low-salinity groups, while *PDH* and *PK* gene expression were only elevated in the seawater-cultured shrimp. These phenomena demonstrated that ammonia stress might induce a metabolic shift from aerobic to anaerobic metabolism in the shrimp intestines, and the seawater-cultured shrimp might sustain energy supply more effectively by enhancing the glycolytic-TCA cycle metabolic flux. The TCA cycle is a process that oxidizes acetyl-CoA from pyruvate breakdown to generate energy [27]. In this study, ammonia stress concurrently down-regulated *MDH*, *CS* and *ODH* gene expression in the shrimp intestine across both groups, while *SDH* expression showed an opposite trend with an increase specific to the seawater-cultured shrimp. Conversely, the low-salinity group exhibited additional suppressions in *IDH* and *FH* genes. These phenomena indicated that ammonia stress interfered with the functional homeostasis of the TCA cycle in shrimp intestine, and the salinity might regulate the expression of different key genes in the TCA cycle, leading to differences in energy metabolism adaptation strategies of the intestine in response to ammonia stress. The respiratory chain serves as the core production line for cellular ATP energy [28]. In this study, following ammonia stress, the expressions of *AtpH*, *CCO*, and *cytC* genes were down-regulated in the shrimp intestine under both culture conditions. Conversely, *NDH* gene expression exhibited a salinity-dependent pattern, with an increase in the seawater group but a decrease in the low-salinity group. These findings indicated that ammonia stress generally inhibited respiratory chain function by downregulating key genes, but the differential expression of *NDH* gene suggested that the shrimp adopt distinct metabolic compensation strategies under different salinity conditions for responding to stress. Therefore, ammonia stress disrupted the energy metabolic homeostasis in the shrimp intestine by affecting the expression of genes encoding critical rate-limiting enzymes in the glycolytic, TCA cycle, and respiratory chain pathways, with the shrimp’s coping strategies differing based on ambient salinity.

A stable microbiota acts as a cornerstone of the host’s biological defenses, forming a barrier that is fundamental to sustaining overall health [29]. Intestinal microbial diversity is closely associated with disease occurrence in shrimp [30]. In this study, ammonia stress consistently increased the ACE, Chao1, and Shannon indices in the shrimp intestine across both salinity groups, demonstrating its role in promoting microbial community diversity, which might be attributed to structural adjustments of the microbial community in response to the stressful environment. Furthermore, the composition of intestinal bacteria was also disturbed by ammonia stress. Proteobacteria contain some pathogenic bacteria, which are recognized as an indicator of intestinal dysbiosis and inflammation [31]. Firmicutes contain certain bacteria that produce beneficial metabolites and possess the ability to regulate the host’s lipid metabolism [32]. In this study, the increase in Proteobacteria and Firmicutes in the shrimp intestine under both salinity conditions indicated that ammonia stress drove the restructuring of core intestinal microbiota under these two salinity conditions. Specifically, the increase in Proteobacteria might contribute to intestinal microecological imbalance, whereas the elevation of Firmicutes could facilitate lipid metabolism and provide beneficial metabolites to the host in adapting to environmental stress. Actinobacteria can produce natural active substances such as antibiotics and exhibit antibacterial capabilities [33]. Tenericutes is a dominant bacterial group in the shrimp intestine [3]. In this study, ammonia stress diminished the abundance of Actinobacteria and Tenericutes in the shrimp intestine across both salinity environments, thereby disrupting the original balance of the intestinal microbiota. This disruption might lead to a reduction in the production of antibacterial active substances in the intestines of the shrimp, weakening the ability to inhibit harmful microorganisms and increasing the risk of infection. Bacteroidetes facilitates the degradation of complex polysaccharides and the subsequent production of short-chain fatty acids (SCFAs), thereby supplying energy to the host and contributing to the regulation of immune function and intestinal homeostasis [34]. In this study, a salinity-dependent shift in intestinal Bacteroidetes abundance was observed after ammonia stress, characterized by an increase in the seawater group and a decrease under low-salinity conditions. This discrepancy suggested that the seawater-cultured shrimp might upregulate Bacteroidetes to enhance energy acquisition and immune regulation as an active response to ammonia stress, whereas the beneficial function of Bacteroidetes in the low-salinity cultured shrimp was inhibited under the dual pressures of salinity and ammonia stress, resulting in a higher health risk for the shrimp.

Ammonia stress also disrupted the homeostasis of several intestinal bacterial genera in shrimp that are potentially critical for host health. *Candidatus Bacilloplasma* typically constitutes a predominant bacterial group within the shrimp intestine, which exhibits frequent population shifts during stress or disease outbreaks with potential negative consequences for host health [35]. In this study, the observed reduction in *Candidatus Bacilloplasma* in both salinity groups suggested that ammonia stress might compromise the stability of the intestinal environment, leading to dysbiosis in this ecologically dominant bacterium, which could further exacerbate the instability of the intestinal microbial community. Furthermore, ammonia stress also disrupts both potentially harmful and beneficial bacterial populations. *Vibrio*, *Tenacibaculum*, and *Staphylococcus* are common opportunistic pathogens in aquaculture [36,37,38]. *Achromobacter* and *Escherichia-Shigella* are opportunistic pathogens whose enrichment is often associated with health abnormalities and an increased risk of infection in both humans and animals [39,40]. In this study, ammonia stress increased the abundances of *Vibrio*, *Tenacibaculum*, *Staphylococcus*, *Achromobacter*, and *Escherichia-Shigella* in both salinity groups. This proliferation of multiple potential pathogens collectively indicated a disruption of intestinal microbiota homeostasis and an elevated risk of infection. As functional bioactive producers, *Bacillus* and *Lactobacillus* are widely applied as probiotics in aquaculture [41,42]; *Alloprevotella, Bacteroides* and *Cetobacterium* metabolize dietary carbohydrates to generate short-chain fatty acids (SCFAs) [43,44,45]; *Demequina* contributes to starch degradation through α-amylase secretion [46]. In this study, following ammonia stress, the shrimp intestine exhibited distinct microbial shifts. A consistent decrease in *Bacillus* and increases in *Bacteroides* and *Cetobacterium* were observed under both salinity conditions. Conversely, *Lactobacillus* and *Demequina* exhibited opposing, salinity-dependent trends. Specifically, *Lactobacillus* increased in seawater but decreased in low salinity, while the inverse pattern was recorded for *Demequina*. Additionally, *Alloprevotella* was uniquely reduced in the low-salinity group. These findings indicated that ammonia stress might disrupt the homeostasis of beneficial functional bacteria in the shrimp intestines, thereby potentially impacting the host’s health. These differential responses of these bacteria to ammonia stress were also affected by the rearing water salinity, suggesting that the tailored strategies for ammonia mitigation and intestinal health management should be adopted according to specific salinity environments.

Furthermore, predicted microbial functions under ammonia stress showed overlapping responses in some pathways between the two groups, while others diverged significantly, reflecting a salinity-dependent effect. A key common response was the significant enhancement of bacterial chemotaxis in both groups, suggesting that ammonia stress might universally trigger intestinal bacteria to enhance their chemotaxis and motility as a general adaptation strategy to environmental stress, irrespective of salinity. Regarding salinity-specific differences, the intestinal microbiota of seawater cultured shrimp demonstrated broad functional enhancement, including upregulated pathways related to carbohydrate metabolism, nitrogen metabolism, vitamin and cofactor metabolism, linoleic acid metabolism, glycosaminoglycan degradation, and bile secretion. This reflected a comprehensive adaptive response aimed at improving nutrient utilization and host physiological regulation under ammonia stress. In contrast, in the low-salinity cultured shrimp, only the PTS function was significantly enhanced, while multiple critical functions, such as protein digestion and absorption, flavonoid and steroid hormone biosynthesis, and glycosaminoglycan degradation, were markedly suppressed. This indicated that the low-salinity environment might exacerbate the functional impairment of intestinal microbiota under ammonia stress, leading to compromised nutrient metabolism and immune regulation. Thus, we deduced that ammonia stress might disrupt the functional profile of the intestinal microbiota of the shrimp in a salinity-dependent manner. The observed functional differences might directly influence the adaptive capacity and nutritional metabolic efficiency of the shrimp reared under different salinity environments.

This study has certain limitations in the establishment of the stress model. Firstly, the ammonia stress employed was only at a single concentration without setting up a concentration gradient, so it is unable to reveal the dose-effect relationship of ammonia toxicity. Secondly, all indicators were measured at a single time point, 14 days after exposure. Although this can provide a static snapshot of intestinal health at that specific time point, it fails to reflect the dynamic changes in the intestine during the stress process, such as early pathological progression or potential recovery ability. This design renders the research conclusions somewhat time-specific. In subsequent studies, setting multiple ammonia concentration gradients and multiple observation time points will help systematically clarify the complete response trajectory of shrimp intestinal health under ammonia stress. In addition, future work can further focus on the toxicological effects of long-term ammonia stress and conduct comprehensive evaluations from dimensions such as growth performance, individual development, overall physiological health, and immune tolerance, to more comprehensively reveal the potential risks of ammonia stress to shrimp.

## 5. Conclusions

This study demonstrated that ammonia stress exerted adverse effects on the intestinal health of *L. vannamei* in both seawater and low-salinity environments. Ammonia stress damaged the intestinal morphological structure of the shrimp under two salinity conditions, inducing oxidative and ER stress, and activating immune, inflammatory, and autophagy responses. It also disrupted intestinal energy metabolism homeostasis by interfering with the expression of key functional genes involved in glycolysis, TCA cycle, and respiratory chain. Furthermore, ammonia stress elevated intestinal microbial diversity, but disrupted microbiota homeostasis by increasing harmful bacteria and reducing beneficial bacteria. These integrated changes collectively impaired the overall intestinal health and functional homeostasis of the shrimp. Although the low-salinity cultured shrimp exhibited certain pre-adaptive adjustments, they demonstrated higher intestinal stress responses and greater physiological vulnerability to ammonia stress when compared to the seawater-cultured shrimp.

## Data Availability

The original contributions presented in this study are included in the article/Supplementary Material. Further inquiries can be directed to the corresponding author.

## Supplementary Materials

The following supporting information can be downloaded at https://www.mdpi.com/article/10.3390/antiox14111383/s1. Table S1: The primer sequences used in this study.
