# Supplementary material for: Ammonia Stress Disrupts Intestinal Health in Litopenaeus vannamei Under Seawater and Low-Salinity Environments by Impairing Mucosal Integrity, Antioxidant Capability, Immunity, Energy Metabolism, and Microbial Community
Source: Antioxidants (Basel). 2025 Nov 20;14(11):1383. doi: 10.3390/antiox14111383 (PMC12649622; doi:10.3390/antiox14111383)
Supplement: Supplementary file 1 [file antioxidants-14-01383-s001.zip › antioxidants-3979635-supplementary.pdf]

Supplementary Materials

Table S1. The primer sequences used in this study.

Gene	Forward primer (5’-3’)	Reverse primer (5’-3’)
SOD	GACACGACCATTAGCCTGTACGAC	CAGCGTTGCCAGTAGCGAGTG
GPx	TCTGAGCGGCGAGATGGTGTC	CTGGTGGAAGTCCCTGGTGGTC
Bip	CCAGCACGACATCCAGTTCTTCC	CCTCCGCAGCAAACACCTTCTC
IRE1	GGTACATTAGGTTCTCGTCCGTCAC	AATTCCTCTGGTGTTGCCTTAGCC
XBP1	CGCCTGCTGAGGATGACCTTATTAC	GCCTACTGGTGATGTGTCCTTAACG
Crus	TGGTGTAGGTGGCGGTCTTGG	CTTGTGGGCAGTCGAGTATCTTGG
Lys	TCGAGTCGTCCTTCAACACG	TGCAGACGTTCTTGCCGTAG
proPO	CAATGACCAGCAGCGTCTTC	CACGGAAGGAGGCGTATCAT
NF-κB	TCTAACCAATCACCACAGCAC	TGGTAAACTCAGTGTTCGGG
Atg3	TGGAGTATCGCAGTGAGCAGGAG	TGCCATGTCAGCCACTTTCTCTTC
Atg12	CGTAAACAATGGAGGGCGAGAAGG	TTCTCCTGCTCCTGGTTTTCTTGTG
Beclin1	GCGACCTACACACAGTATGCCTAAC	CCATCCTCCATGCTCTCCAACAAG
PDH	TCAGCCTCAACCAACTACTACACTC	GCCTCCTTCACACTCAGTACATCC
HK	ACCTGCTGCTGGTTCACGATG	GCTGCTGCCTCCTCCAAGTG
PK	GCCAGACAGTGCCATCTCTACC	TGCCAGCCAGTCACCACAAC
LDH	GATCGGCTCAGGCACCAACC	GCAACATTAACACCAGACCAGACAG
MDH	CTCTTCCACCCAGTTCCCAGATG	GACAACTTACGAGCGGCAATGAC
CS	GCTCGGTTCCATCCATCCTCTG	ACGCCTTCTGTTGGTGTCCTAAG
SDH	TTCCTGGCACTCACTATGACTGTTC	GATGAAGTAGCAGAGACCTCCCAAG
IDH	ACGGAGACCAATACAAGGCTACTG	TGAGTGTGCGAAGGAACGGATAG
FH	TTGTTCCTCTTCTCGCCTGGTTAC	TCTGACTCCATCTTTGCCACTCTTC
ODH	GCAAGGCATAATCAGGGCATATCAG	CATAGGAGCGGACTACTGTTCTGG
NDH	CTTCTTGGTTCGGTGCTTGAATGG	AGCAGCCTCTGAAGAGTATTGGTTG
AtpH	CACCATCATCAACCAGAAGCGATTC	GGAGCAGCATCAGAGGCAGTG
CCO	ATGCCAGGTGTCCGCTTCAAG	AAGGGTCAACTTGTTCCAGTCTCC
cytC	TCGACGTGTACCTGACCAACCC	TTCGCCTGGCTTCCTCTTCCTC
β-actin	TCGCTCCCTCCACCATGAAGATC	CTCCTGCTTGCTGATCCACATCTG
